# Rheological Behavior of Crumb Rubber/Plasticized Crumb Rubber Composite Modified Asphalts Under Different Aging Conditions

**DOI:** 10.3390/ma19214449

**Published:** 2026-10-22

**Authors:** Pengfei Qing, Peng Xu, Junwu Xi, Qian Bu, Xinzhao Dong, Qian Liu, Chenxu Zhu

**Affiliations:** 1 Maintenance Engineering Company of ShaanXi Road and Bridge Group Co., Ltd., Xi’an 710065, China; qpf123@126.com (P.Q.); 13572522034@163.com (J.X.); 15249270228@163.com (Q.L.); 2 Xi’an Huaze Road Material Co., Ltd., Xi’an 710061, China; bbq7211@163.com; 3 School of Materials Science and Engineering, Chang’an University, Xi’an 710018, China

**Keywords:** road engineering, crumb rubber/plasticized crumb rubber composite modified asphalt, short-term aging, long-term aging, rheology

## Abstract

Composite modified asphalt was prepared from SK90# base asphalt, 40-mesh ordinary crumb rubber, plasticized crumb rubber, and TOR to study its rheological aging behavior. TFOT and PAV simulated short- and long-term aging, while physical tests, temperature sweep, and MSCR tests by DSR analyzed the influencing factors. Crumb rubber strengthens asphalt hardness and high-temperature deformation resistance. Plasticization improves rubber–asphalt compatibility and reduces viscosity, complex modulus, and rutting factor. Under the tested aging conditions, the plasticized crumb rubber-modified asphalt shows higher aging sensitivity in high-temperature rheological and MSCR indicators. Rising stress lowers the recovery rate and elevates non-recoverable creep compliance. TOR optimizes aged rheology, and composite modified asphalt outperforms single rubber-modified asphalt after long-term aging.

## 1. Introduction

Heavy traffic load is a key factor affecting the durability of asphalt pavements. Crumb rubber-modified asphalt has been widely used in pavement engineering due to its excellent road performance [1,2]. Currently, most scholars investigate the evolution trends in the performance of crumb rubber-modified asphalt under the effects of indoor high temperature, ultraviolet irradiation, and other factors to improve the service quality and extend the service life of crumb rubber-modified asphalt pavements [3,4].

To date, considerable progress has been made in research on the performance of crumb rubber-modified asphalt. Many scholars have investigated the pavement performance of crumb rubber-modified asphalt (CRMA) from the perspectives of dosage, chemical structure, and released substances, and have explored the factors affecting asphalt performance from the crumb rubber itself [1,5,6]. Through these studies, it has been found that appropriate pretreatment methods and combination with other materials can further increase the crumb rubber content and improve the pavement performance [7,8,9]. However, in actual service, the aging behavior of CRMA is also a key factor affecting its service life: Chen et al. focused on the effect of short-term aging on the high-temperature performance of the rubber-modified asphalt, pointing out that the addition of crumb rubber can greatly improve the high-temperature performance of the asphalt [10]. Gao et al. concluded that aging has only a slight effect on the rheological properties of the crumb rubber-modified asphalt, and the addition of crumb rubber improves the aging resistance of the asphalt [11]. Wang et al. studied the rheological behavior of high-content crumb rubber-modified asphalt before and after aging, and concluded that the elastic component of the crumb rubber-modified asphalt is positively correlated with the aging degree [12]. Although the addition of crumb rubber can improve the road performance of base asphalt, its compatibility with asphalt is also considered the main factor limiting its dosage. Xie et al. prepared graft-activated crumb rubber and found that the activated crumb rubber is beneficial for alleviating the formation of heavy components [13]. Li et al. solved the problems of poor compatibility and thermal stability between crumb rubber and asphalt by microwave activation, and found that the activated crumb rubber-modified asphalt has good aging resistance [14].

To address the compatibility issue of crumb rubber-modified asphalt, scholars have attempted to incorporate TOR as a linking agent. Li et al. found that the TOR linking agent promoted the dissolution of surface-treated crumb rubber in asphalt, thereby improving compatibility [15]. Liang et al. experimentally found that the participation of the double bonds in TOR in chemical reactions contributes to stability, thus improving the compatibility and storage stability of crumb rubber-modified asphalt [16]. Zhang et al. used TOR to improve the interfacial compatibility between the elastomer system and the base asphalt [17]. However, existing studies have not sufficiently investigated its behavior during asphalt aging.

Therefore, based on current research, this study investigates the effects of unactivated crumb rubber, activated crumb rubber, and TOR linking agent on base asphalt when used alone and in combination. In addition, considering that current studies are still insufficient regarding the evolution behavior of TOR/crumb rubber-modified asphalt under aging, SK90# base asphalt was used as the raw material, and 40-mesh ordinary crumb rubber, plasticized crumb rubber, and Vestenamer (TOR) were used as additives to prepare composite modified asphalt with different proportions. Combined with physical properties, temperature sweep (TS), and multiple stress creep recovery (MSCR) tests, the rheological behavior of the crumb rubber/plasticized crumb rubber composite modified asphalt under different aging degrees was systematically studied. The effects of crumb rubber type, additive, and aging degree on the rheological indexes of the composite modified asphalt were analyzed to provide a technical basis and reference for improving the road performance of crumb rubber-modified asphalt.

## 2. Materials and Methods

### 2.1. Raw Materials

**Figure 1:**
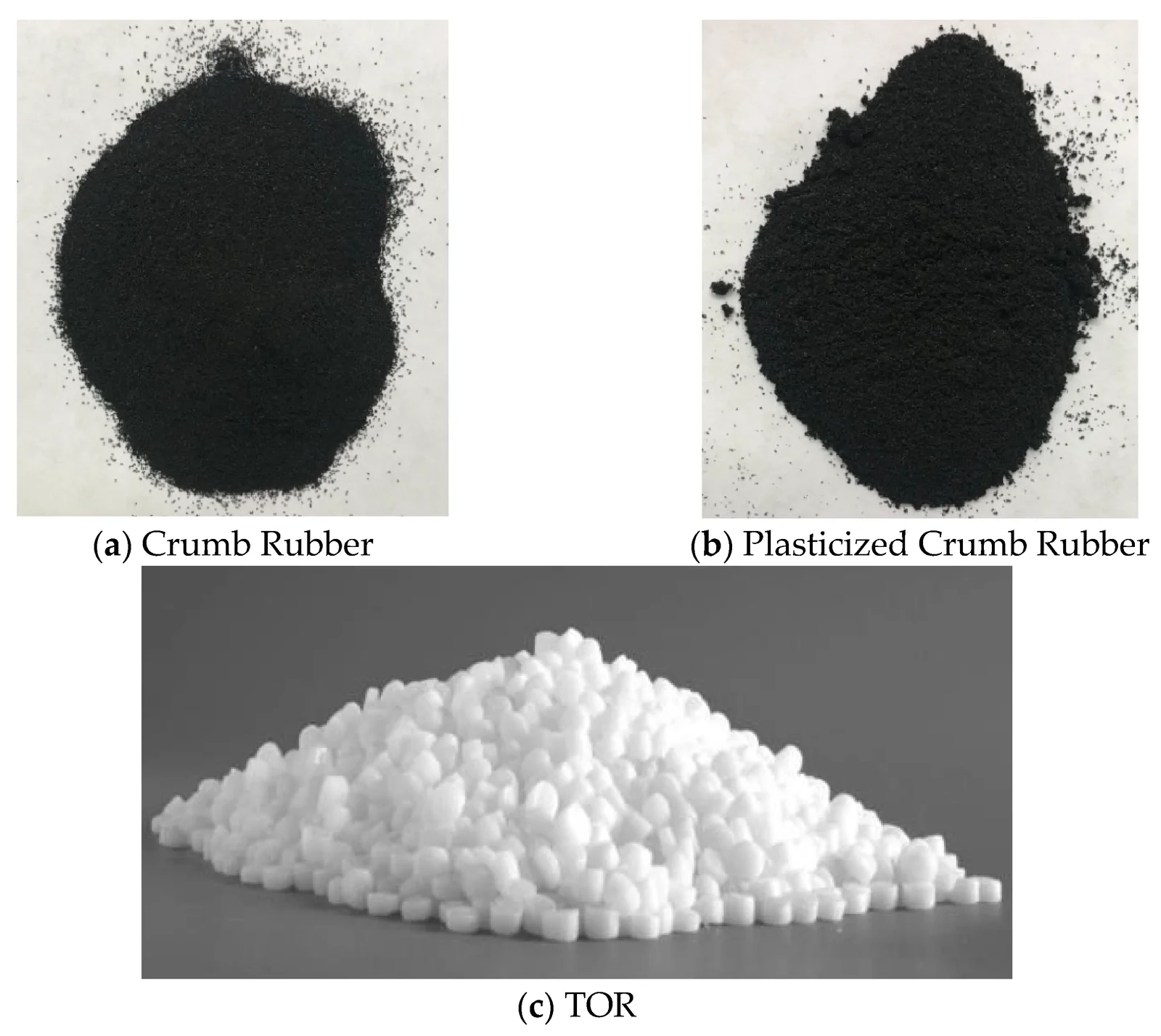
Appearances of crumb rubber, plasticized crumb rubber, and TOR.

The crumb rubber used is produced by CCCC Banana Resource Recycling Co., Ltd. (Qingdao, China), and the plasticized crumb rubber was prepared from crumb rubber of the same particle size by atmospheric-pressure plasticization in a twin-screw extruder (Shandong Dongsheng Rubber Technology Co., Ltd, Rizhao, China), with the screw speed controlled at 50 r/min and the barrel temperature maintained at 220 °C. The additive used was Vestenamer^®^ 8012 (TOR), which is a trans-polyoctenamer rubber, provided by Evonik Specialty Chemicals Co., Ltd. (Shanghai, China). The technical indexes for the SK90# base asphalt and crumb rubber used in this test are shown in Table 1, and the technical indexes for TOR are shown in Table 2. The appearances of crumb rubber, plasticized crumb rubber, and TOR are shown in Figure 1.

### 2.2. Preparation of Composite Modified Asphalts

Composite modified asphalt specimens are characterized by the mass and type of admixtures. When the dosage of the ordinary crumb rubber (40 mesh) was 20%, 10%, and 0%, respectively, the dosage of the plasticized crumb rubber (40 mesh) was 0%, 10%, and 20%, respectively. The total external proportion of crumb rubber was 20% of the mass of the base asphalt. The dosage of TOR was 5% of the total mass of crumb rubber. The preparation steps of the modified asphalt are as follows:

(1) The SK90# base asphalt was heated to 180 °C to make it flowable, then crumb rubber/plasticized crumb rubber and TOR were added evenly and stirred at 500 rpm until the crumb rubber and TOR were completely mixed.
(2) The mixture was high-speed sheared at 5000 rpm for 45 min at 180 °C, and the composite modified asphalt was kept in an oven at 180 °C for 45 min for development.

In the subsequent tests, unaged specimens were named by the type and dosage of admixtures. For example, the specimen with 20% crumb rubber was recorded as J20, and the specimen with 10% crumb rubber and 10% plasticized crumb rubber was recorded as (J10 + S10). Aged specimens were named by the aging mode, type, and admixture dosage. For example, the specimen with 20% crumb rubber after TFOT aging was recorded as T(J20), and the specimen with 10% crumb rubber and 10% plasticized crumb rubber after PAV aging was recorded as P(J10 + S10).

### 2.3. Tests

#### 2.3.1. Asphalt Aging Test

The asphalt aging tests were conducted in accordance with JTG 3410-2025 (Standard Test Methods of Asphalt and Asphalt Mixture for Highway Engineering, Publisher: Ministry of Transport of the People’s Republic of China: Beijing, China, 2025) [18] and included TFOT and pressure aging vessel test (PAV). Specifically, the TFOT test involves placing a sample pan containing 50 g of sample into a thin-film oven (Suzhou Tainuo Oven Manufacturing Co., Ltd., Suzhou, China) at 163 °C for 5 h. For the PAV test, the asphalt sample is first placed in a pressure vessel at 100 °C. An air pressure of 2.1 MPa ± 0.1 MPa is then applied and maintained for 20 h. Subsequently, the sample is placed in an electrically heated forced-air drying oven (Suzhou Tainuo Oven Manufacturing Co., Ltd., Suzhou, China) at 168 °C ± 5 °C and heated for 15 min ± 1 min, while a sample-pouring container is preheated to 168 original article ±5 °C. After the asphalt sample has been transferred into the sample-pouring container, the container is placed in a vacuum drying vessel at 170 °C ± 5 °C and heated for 15 min ± 1 min. The pressure is then reduced until the negative pressure reaches 15 kPa ± 2.5 kPa, and this pressure is held for 30 min ± 1 min, yielding PAV-aged asphalt.

#### 2.3.2. Conventional Properties of Asphalt

The physical property tests, including 25 °C penetration, softening point, 5 °C ductility, viscosity, and mass loss of the crumb rubber-modified asphalt, were conducted in accordance with JTG 3410-2025 to evaluate the macroscopic performance of the crumb rubber/plasticized crumb rubber composite modified asphalt.

#### 2.3.3. Rheological Property Test

A dynamic shear rheometer (DSR, Anton Paar SmartPave 102, Shanghai, China) was used to conduct TS and MSCR tests to measure the rheological properties of the crumb rubber/plasticized crumb rubber composite modified asphalt under unaged, short-term aged, and long-term aged conditions. The rheological tests in this study used a parallel-plate geometry with a plate diameter of 25 mm ± 0.5 mm and a gap of 1 mm. For the TS test, the frequency was 10 rad/s, the strain level was 12%, and the temperature range was 20–90 °C. Considering the complexity of the data, 30 °C, 50 °C, 70 °C, and 90 °C were selected for rheological characterization in this analysis. The MSCR test was conducted at 64 °C: a 0.1 kPa load was applied for 1 s and then unloaded for 9 s, forming one cycle; this cycle was repeated 10 times, followed by the same procedure under a 3.2 kPa load. Only the data from the cyclic phase were analyzed and are presented in this paper. The creep recovery rate (*R*) and non-recoverable compliance (*J*_nr_) of the asphalt samples were determined under different stress levels.

## 3. Results and Discussion

### 3.1. Fourier Transform Infrared Spectroscopy (FT-IR)

**Figure 2:**
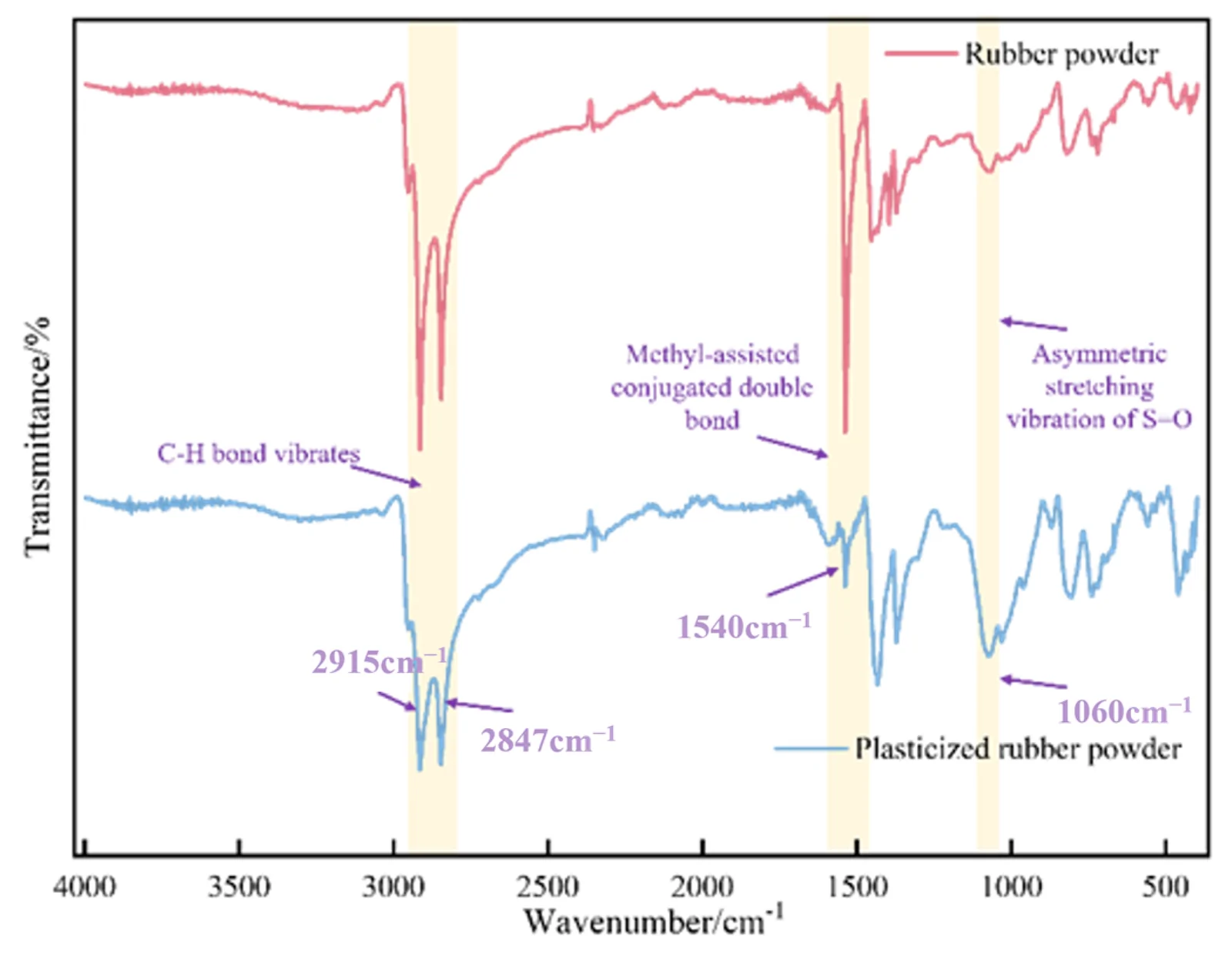
FT-IR spectra of the two types of crumb rubber.

The FT-IR test results for the crumb rubber samples are presented in Figure 2. From the FT-IR spectra of the two types of crumb rubber, no new characteristic peaks appeared after plasticization, indicating that plasticization is a physical process. Prominent absorption peaks were observed at 2915 and 2847 cm^−1^, which were mainly attributed to the C-H stretching vibrations of methylene (-CH_2_) groups, indicating that the main components are hydrocarbons. The absorption peak at 1540 cm^−1^ is typically observed in vulcanized natural rubber. In the plasticized crumb rubber, the intensity of this characteristic peak was substantially reduced compared with that of the crumb rubber, suggesting that devulcanization may have occurred, the stable cross-linked structure was disrupted, and the degree of activation increased. In addition, the peak intensity of the plasticized crumb rubber at 1060 cm^−1^ increased, indicating that an oxidation reaction occurred during plasticization.

**Figure 3:**
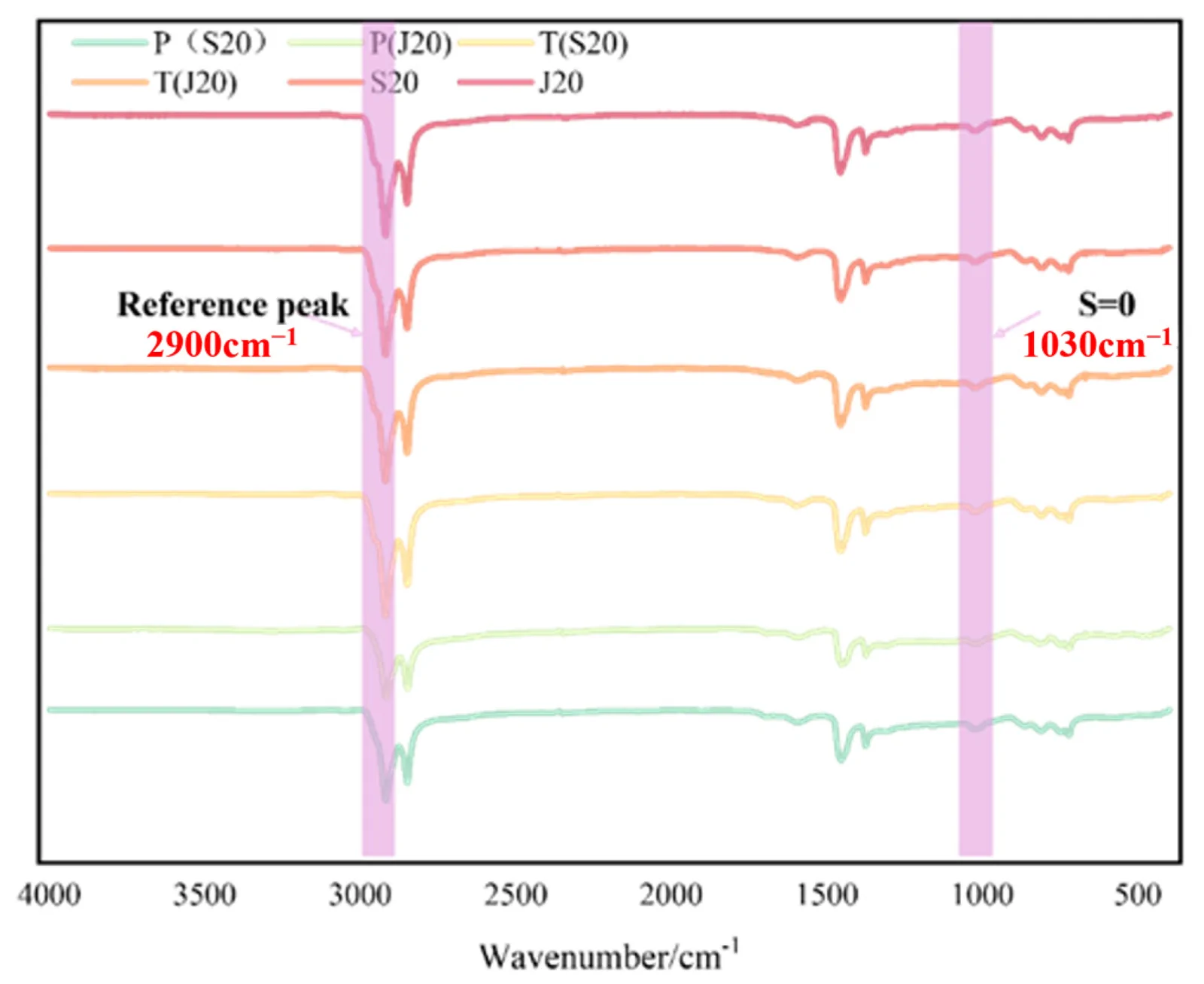
Infrared spectra of crumb rubber asphalt and plasticized crumb rubber asphalt under different aging conditions.

Meanwhile, we also conducted infrared spectroscopy tests on crumb rubber asphalt and plasticized crumb rubber asphalt, and the results are shown in Figure 3. The sulfoxide group (S=O) was used as the criterion for evaluating the degree of oxidation, and the specific calculation method is given in Equations (1) and (2). The detailed results are presented in Table 3.

$$\mathrm{SI}=\frac{A_{S=0}}{A_{R}}$$

where *SI* is the sulfoxide index, *A_S=O_* is the sulfoxide peak area, and *A_R_* is the reference peak area.

From Table 3, the sulfoxide index of plasticized crumb rubber-modified asphalt is always higher than that of crumb rubber-modified asphalt. This may be because the stable cross-linked structure of the crumb rubber is destroyed after plasticization, thereby increasing its reactivity with oxygen. It can therefore be inferred that plasticization reduces long-term aging resistance.

### 3.2. Physical Property Test

**Figure 4:**
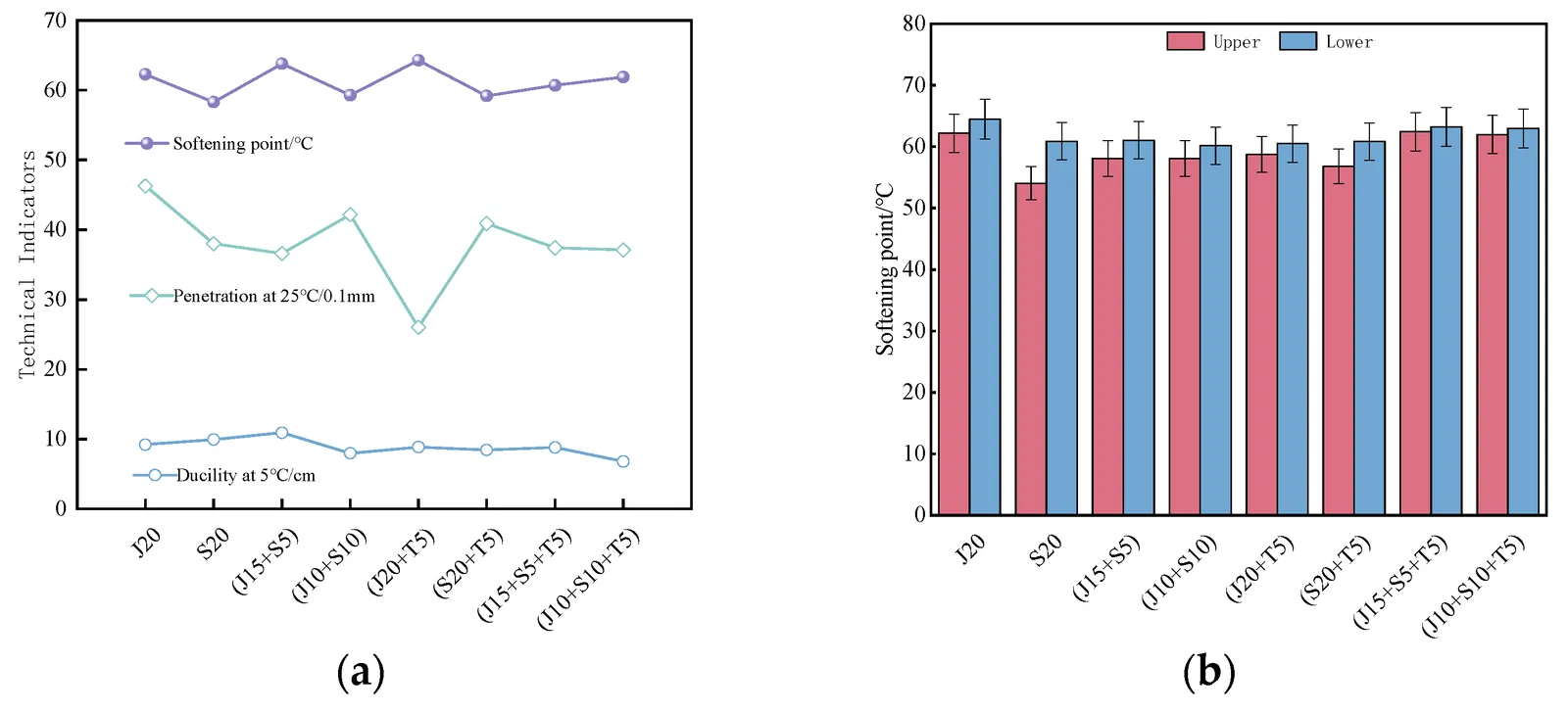

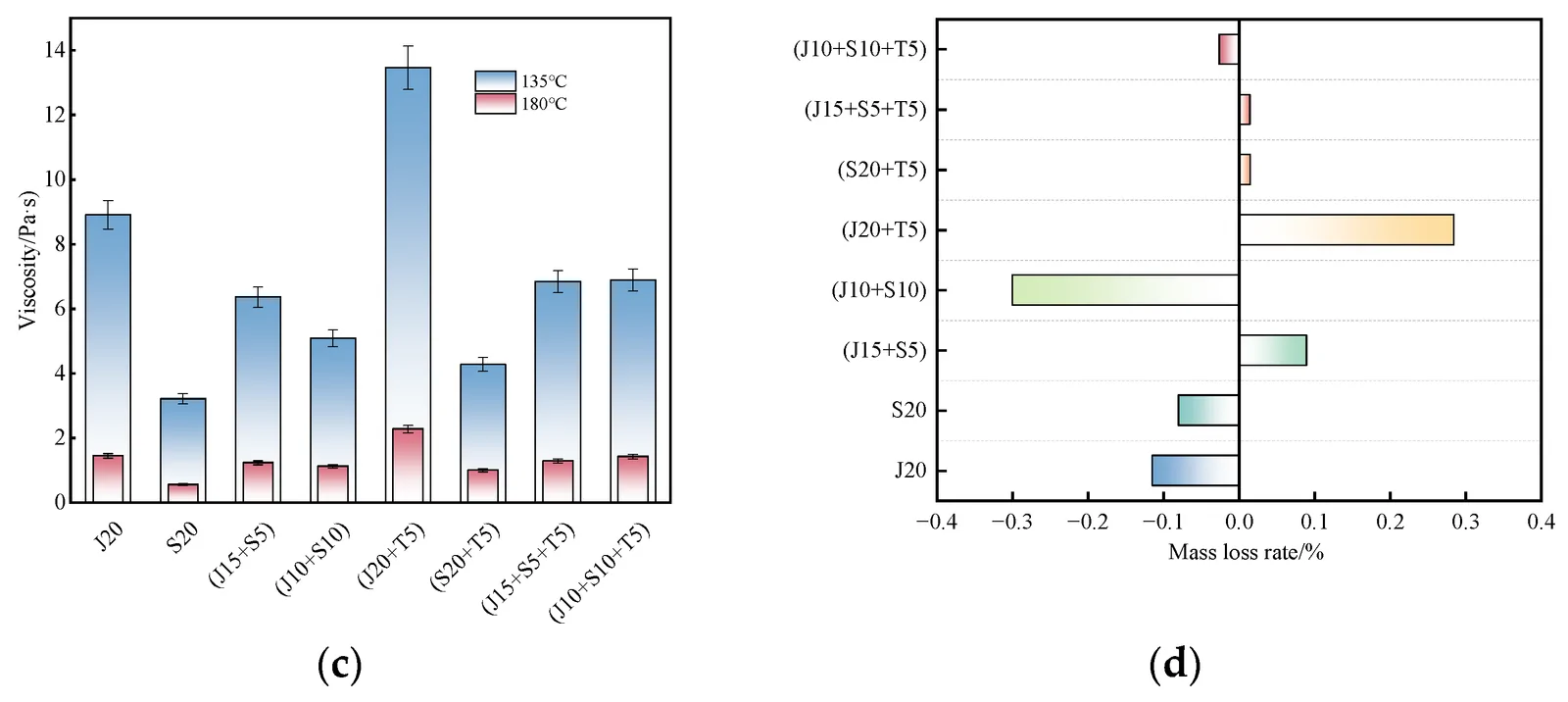
Performance of the composite modified asphalt. (**a**) Basic performance indicators of modified asphalt; (**b**) Softening-point difference after segregation; (**c**) Viscosity; (**d**) Mass loss after thin film oven test (TFOT) aging.

The results of the physical property test of the composite modified asphalt are shown in Figure 4. The softening-point difference and viscosity are technical indexes to characterize the workability and flexibility of asphalt [15,19,20]. The mass change in asphalt after TFOT can be used to evaluate the short-term aging resistance.

According to the penetration, softening point, and ductility test results in Figure 4a, the addition of crumb rubber decreased penetration and increased softening point, indicating increased consistency and improved softening resistance of the asphalt. After plasticization treatment, the compatibility between the crumb rubber and asphalt was improved, and the 5 °C ductility increased, indicating improved low-temperature extensibility. The softening-point results also indicated that the softening resistance of the plasticized crumb rubber-modified asphalt decreased under the softening-point test conditions. The addition of TOR increased the softening point of the composite modified asphalt. This phenomenon is attributed to the inherent hardness of TOR, and within the measured indicators, the high-temperature softening resistance of the composite modified asphalt was improved. In the softening-point difference test, J20 exhibited an upper–lower softening-point difference of 2.3 °C, whereas that of S20 increased to 6.9 °C, indicating poorer storage stability of S20. After composite blending of ordinary crumb rubber and plasticized crumb rubber and the introduction of TOR, the upper–lower softening-point difference of the composite modified asphalt decreased by 85.5–88.4% compared with that of S20, indicating improved macroscopic storage stability under this test condition. This phenomenon may be related to the regulating effect of TOR on the crumb rubber–asphalt interface and network structure. The viscosities of J20 at 135 °C and 180 °C were 8.91 Pa·s and 1.443 Pa·s, respectively. The viscosity of S20 was significantly reduced, with the two indicators decreasing by 63.94% and 61.61%, respectively, indicating that plasticization effectively reduced the system viscosity and improved workability. After composite blending, the viscosities of (J15 + S5) and (J10 + S10) were lower than that of J20 but significantly higher than that of S20. The viscosity of (J20 + T5) with TOR increased sharply, and the viscosities at 135 °C and 180 °C increased by 51.14% and 57.52%, respectively, compared with J20, indicating that TOR can significantly increase the high-temperature viscosity of crumb rubber asphalt but deteriorates its workability. The viscosities of (J15 + S5 + T5) and (J10 + S10 + T5) were slightly lower than those of the crumb rubber-modified asphalt but significantly higher than those of the plasticized crumb rubber asphalt, indicating that TOR can effectively compensate for the high-temperature viscosity attenuation caused by plasticization and balance high-temperature performance and workability in terms of the measured viscosity indicators. As shown in Figure 4d, the absolute values of the mass loss rates of all specimens were lower than those of the original base asphalt, but the mass loss during short-term aging fluctuated with changes in crumb rubber type, dosage, and TOR. The mass loss rate of the plasticized crumb rubber-modified asphalt after aging was lower than that of the ordinary crumb rubber, indicating that crumb rubber activation after plasticization may be more conducive to inhibiting the volatilization of light components; however, mass loss rate only reflects mass change and cannot be directly equated with aging resistance.

### 3.3. TS Test

**Figure 5:**
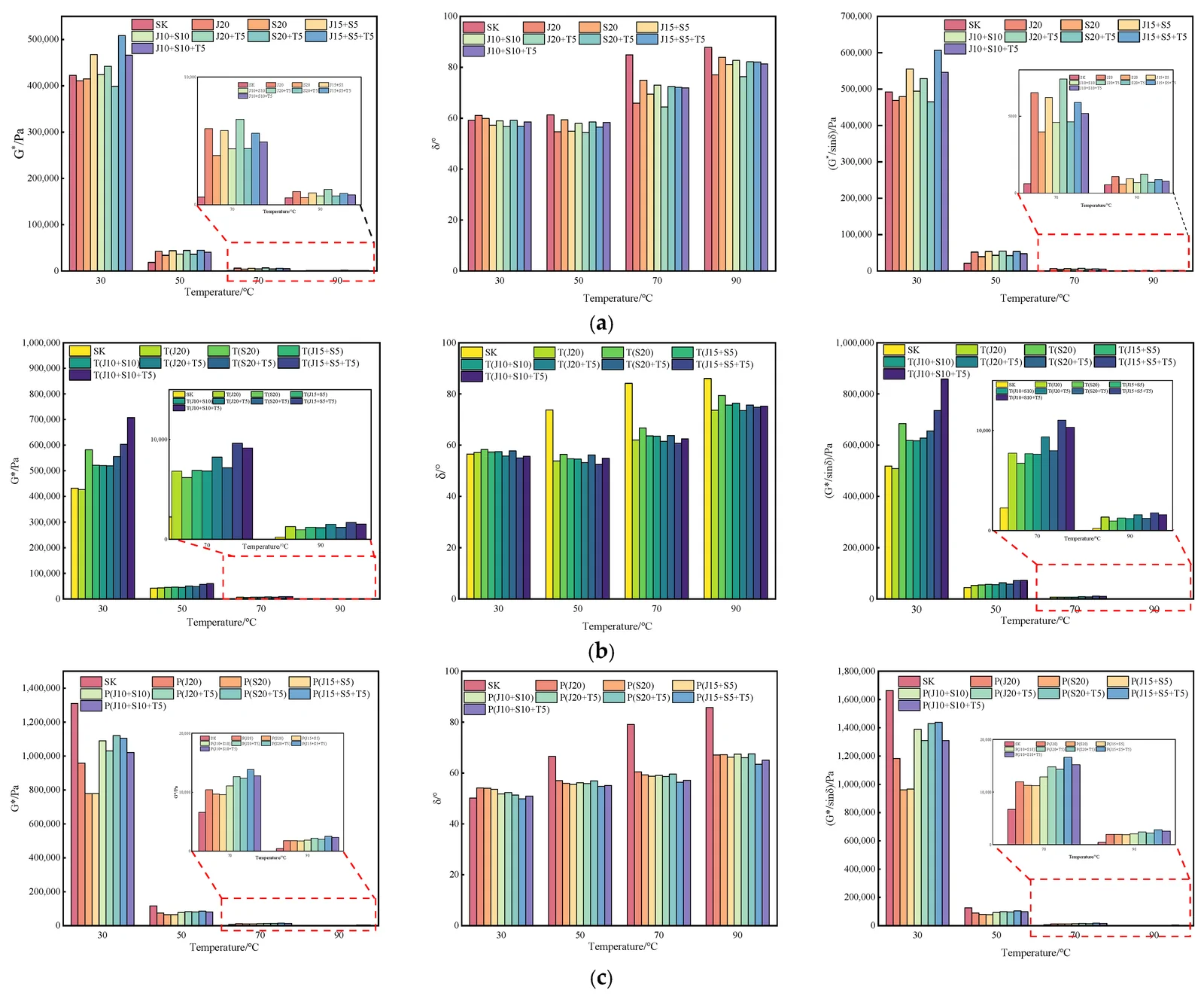

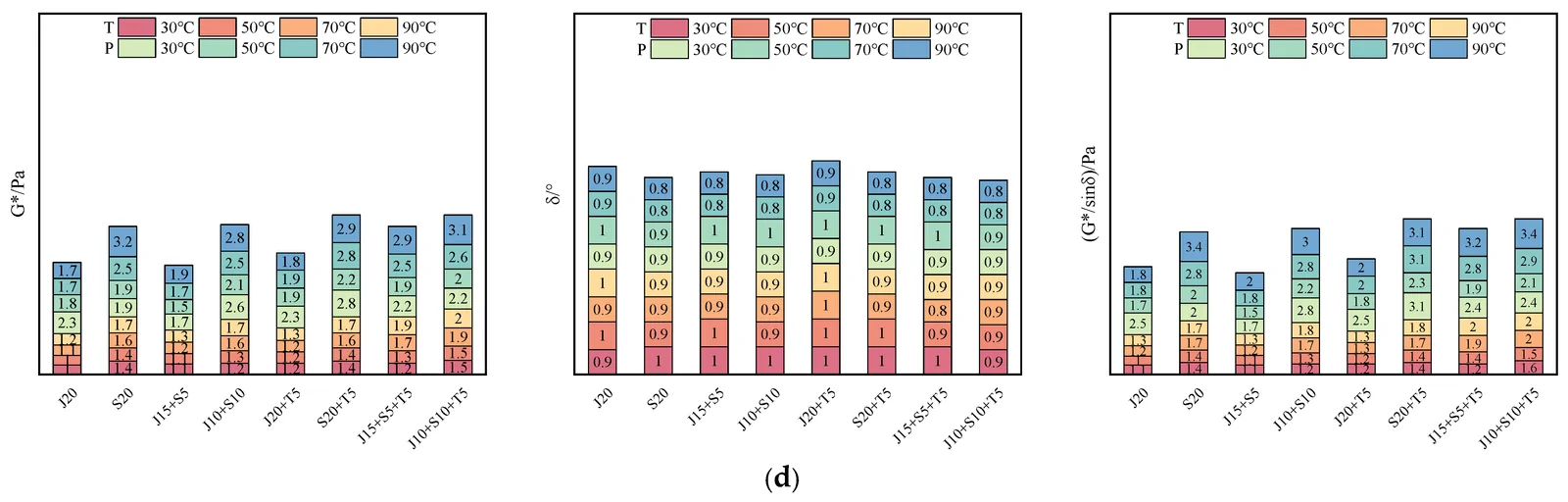
Temperature scanning test for composite modified asphalt. (**a**) G*, δ, G*/sinδ of unaged composite modified asphalt; (**b**) G*, δ, G*/sinδ of short-term aging asphalt; (**c**) G*, δ, G*/sinδ of long-term aging asphalt; (**d**) Changes in G*, δ, G*/sinδ of composite modified asphalt under different aging conditions.

The TS mode was used to obtain the complex modulus (G*), phase angle (δ), and rutting factor (G*/sinδ) of the composite modified asphalt under different aging degrees, to evaluate their effect on the deformation resistance of the crumb rubber-modified asphalt [21,22]. The testing temperature ranged from 20 to 90 °C in 1 °C increments. Due to data redundancy, 30, 50, 70, and 90 °C were selected from the continuous temperature range for analysis. The test results are shown in Figure 5a–c. For convenient comparison, the three technical indexes after aging were quantified by the ratio to those before aging, and the results are shown in Figure 5d. The calculation method is shown in Equation (1):

∆=*A_aged_*/*A_virgin_* (1)

where: ∆ is the rate of change, A_aged_ is the corresponding value of the aged asphalt, and A_virgin_ is the corresponding value of the unaged asphalt.

The TS test (Figure 5) shows that the complex modulus (G*) and G*/sinδ of all specimens decreased with increasing temperature. As shown in the figure, the incorporation of crumb rubber enhances the high-temperature performance of the base asphalt. The G* of the crumb rubber-modified asphalt (J20) decreased by 97.4% during heating, indicating that increasing temperature reduced the proportion of elastic components and deteriorated the high-temperature rheological response of the modified asphalt. In addition, an increase in G*/sinδ also indicates a higher contribution of high-temperature elastic components under the test conditions.

In the case of single doping with ordinary crumb rubber or plasticized crumb rubber, the G* and G*/sinδ of the crumb rubber-modified asphalt showed different trends with aging time. At 30 °C, G* and G*/sinδ first increased and then decreased with increasing aging time. At 50 °C, G* and G*/sinδ increased with aging time and remained stable during long-term aging. Above 50 °C, G* and G*/sinδ gradually increased with aging time. The G* and G*/sinδ of the plasticized crumb rubber-modified asphalt increased with aging time at different temperatures. In short-term aging, the G* of the crumb rubber-modified asphalt decreased by 99.7%, and that of the plasticized crumb rubber asphalt decreased by 99.84%, whereas the G* of (J10 + S10), (J20 + T5), (S20 + T5), (J15 + S5 + T5), and (J10 + S10 + T5) in the composite system decreased by 99.71%, 99.78%, 99.78%, 99.72%, and 99.78%, respectively. These results, based on G* and G*/sinδ, indicate that the high-temperature rheological parameters of the plasticized crumb rubber-modified asphalt have higher aging sensitivity, especially after long-term aging. This phenomenon occurs because the stable cross-linking structure of the crumb rubber is destroyed during plasticization, causing the plasticized crumb rubber to readily react with external oxygen, which may be the reason for the weakening of its long-term aging resistance.

When the two types of crumb rubber are mixed, the G* and G*/sinδ of the unaged modified asphalt increase, but the extent of this increase decreases with increasing plasticized crumb rubber content. With increasing temperature, G* and G*/sinδ gradually become lower than those of the crumb rubber-modified asphalt but remain higher than those of the plasticized crumb rubber-modified asphalt. In addition, with increasing aging time, the change in G*/sinδ of the mixed crumb rubber-modified asphalt is greater than that of the crumb rubber-modified asphalt but still smaller than that of the plasticized crumb rubber-modified asphalt. When TOR is added to the crumb rubber-modified asphalt, its macroscopic hardness is further improved, resulting in increases in G* and G*/sinδ; this trend remains with increasing temperature and aging time. When TOR is added to the plasticized crumb rubber-modified asphalt, although the compatibility between the plasticized crumb rubber and the base asphalt is further enhanced, the hardness of TOR itself increases G* and G*/sinδ. Based on the G*/sinδ results, TOR does not change the degree of elastic contribution of the crumb rubber asphalt under short-term aging, but it increases the extent of its change under long-term aging. When the two types of crumb rubber are mixed with TOR, TOR increases the change in G*/sinδ of the modified asphalt, indicating greater aging sensitivity based on this parameter. With the addition of TOR, the G* and G*/sinδ of the unaged composite crumb rubber-modified asphalt show different results at different temperatures. Overall, the plasticized crumb rubber delays the increase in the contribution of elastic components during aging, but the hardness of TOR itself enhances the resistance of the crumb rubber-modified asphalt to external forces.

### 3.4. MSCR Test

**Figure 6:**
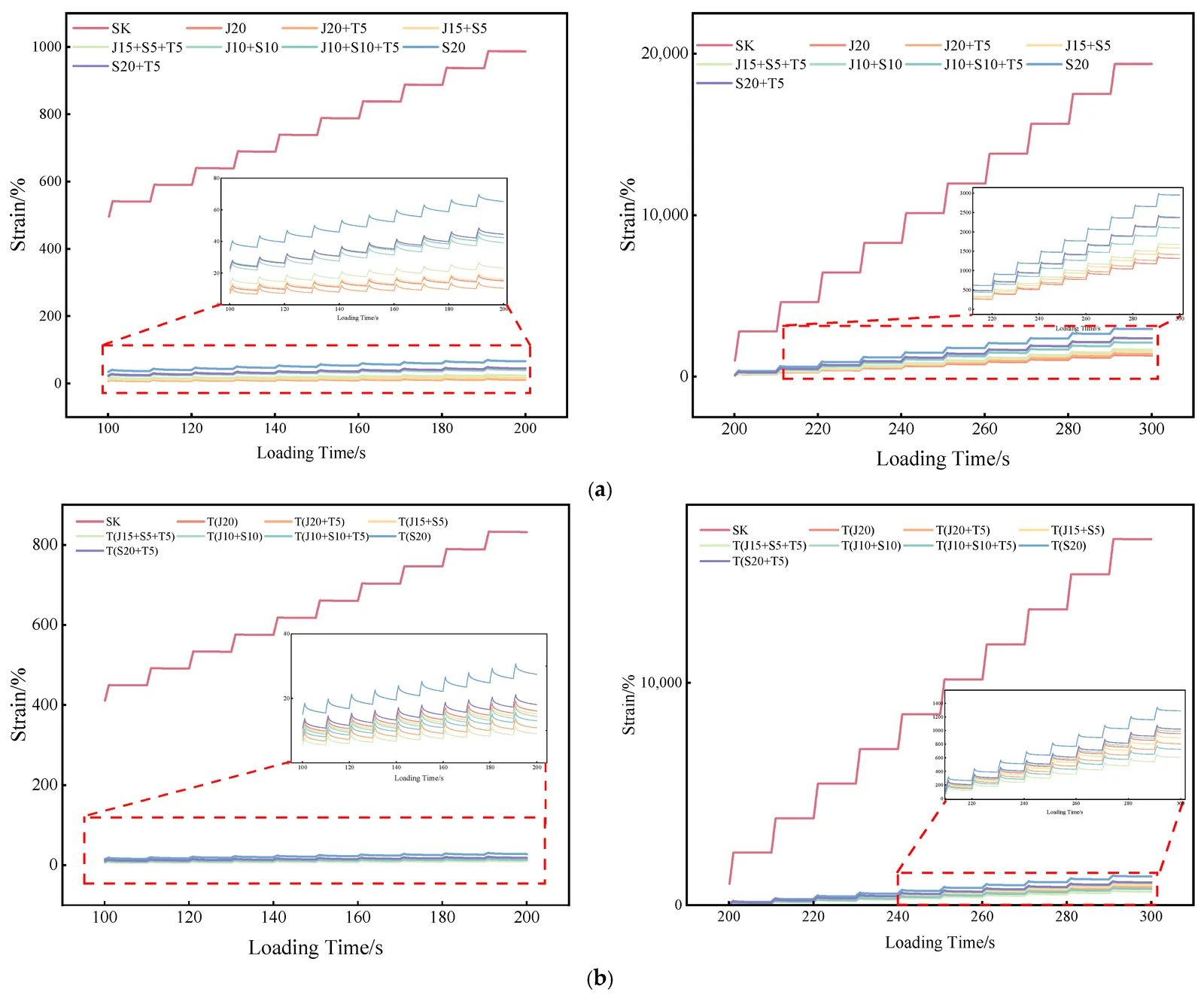

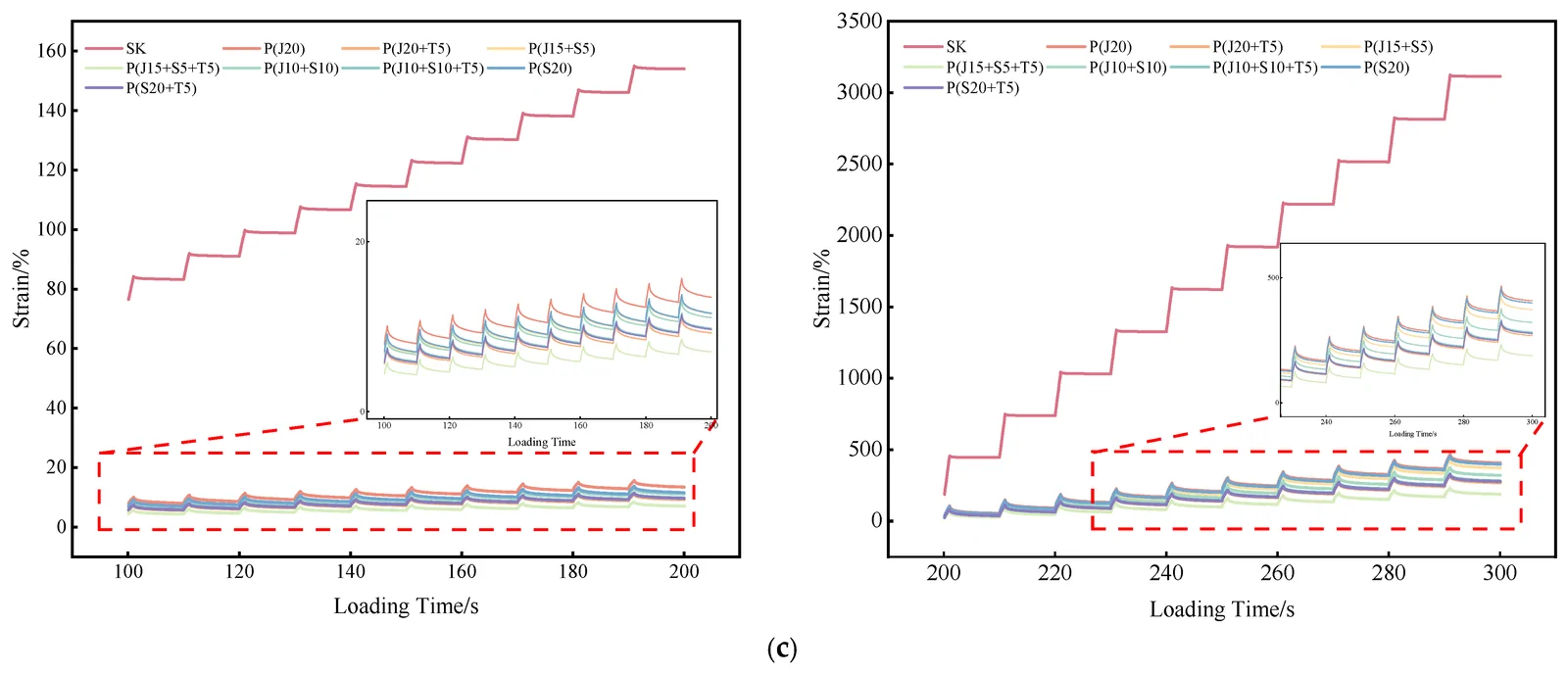
Multiple stress creep and recovery (MSCR) test results of composite modified asphalt. (**a**) MSCR test of unaged composite modified asphalt; (**b**) MSCR test of short-term aged composite modified asphalt; (**c**) MSCR test of long-term aged composite modified asphalt.

The MSCR test explores the deformation resistance of asphalt under different stresses in intermittent loading mode [23,24,25]. The results are shown in Figure 6.

Figure 6 shows the strain accumulation of the crumb rubber asphalt with different aging degrees under two stresses, 0.1 kPa and 3.2 kPa. It can be seen that, under the same conditions, the strain of the base asphalt is much greater than that of the crumb rubber-modified asphalt. The cumulative strain of the crumb rubber-modified asphalt increased significantly with the increase in the stress level, indicating that the binder is strongly stress-sensitive under the MSCR test conditions. After plasticization, the shear resistance of the plasticized crumb rubber asphalt was lower than that of the crumb rubber asphalt, because the plasticized crumb rubber enhanced the compatibility with asphalt and reduced the effect on the asphalt stiffness. When crumb rubber and plasticized crumb rubber were mixed with TOR under unaged conditions, the addition of TOR improved the shear resistance of the crumb rubber asphalt under low stress levels and deteriorated the shear performance under high stress levels. For the plasticized crumb rubber asphalt, the addition of TOR improved the shear resistance under different stress levels. In the case of the composite blending of the crumb rubber and plasticized crumb rubber, the shear performance of the modified asphalt weakened with the increase in the dosage of plasticized crumb rubber. Under low stress level, TOR improved the compatibility between the composite crumb rubber and asphalt, resulting in poor shear performance. However, under a high stress level, TOR improved the shear performance with the increase in the dosage of plasticized crumb rubber.

With increasing aging degree, the crumb rubber asphalt exhibited higher *R*_3.2_ and lower *J*_nr3.2_ under the MSCR test conditions, corresponding to higher elastic recovery and lower non-recoverable compliance at the test temperature. These changes are consistent with binder hardening and an increased elastic or solid-like response after aging. The trend in strain change with time for each aged specimen was the same as that of the unaged specimen. After short-term aging, with increasing loading time, the difference between the plasticized crumb rubber asphalt and other specimens increased significantly, indicating that its rate of residual strain accumulation was significantly higher than that of other specimens. After long-term aging, the differences among all specimens decreased within the test loading time under 3.2 kPa stress. Overall, these MSCR results should be interpreted as indicators of the stress-dependent permanent deformation response at the test temperature.

**Figure 7:**
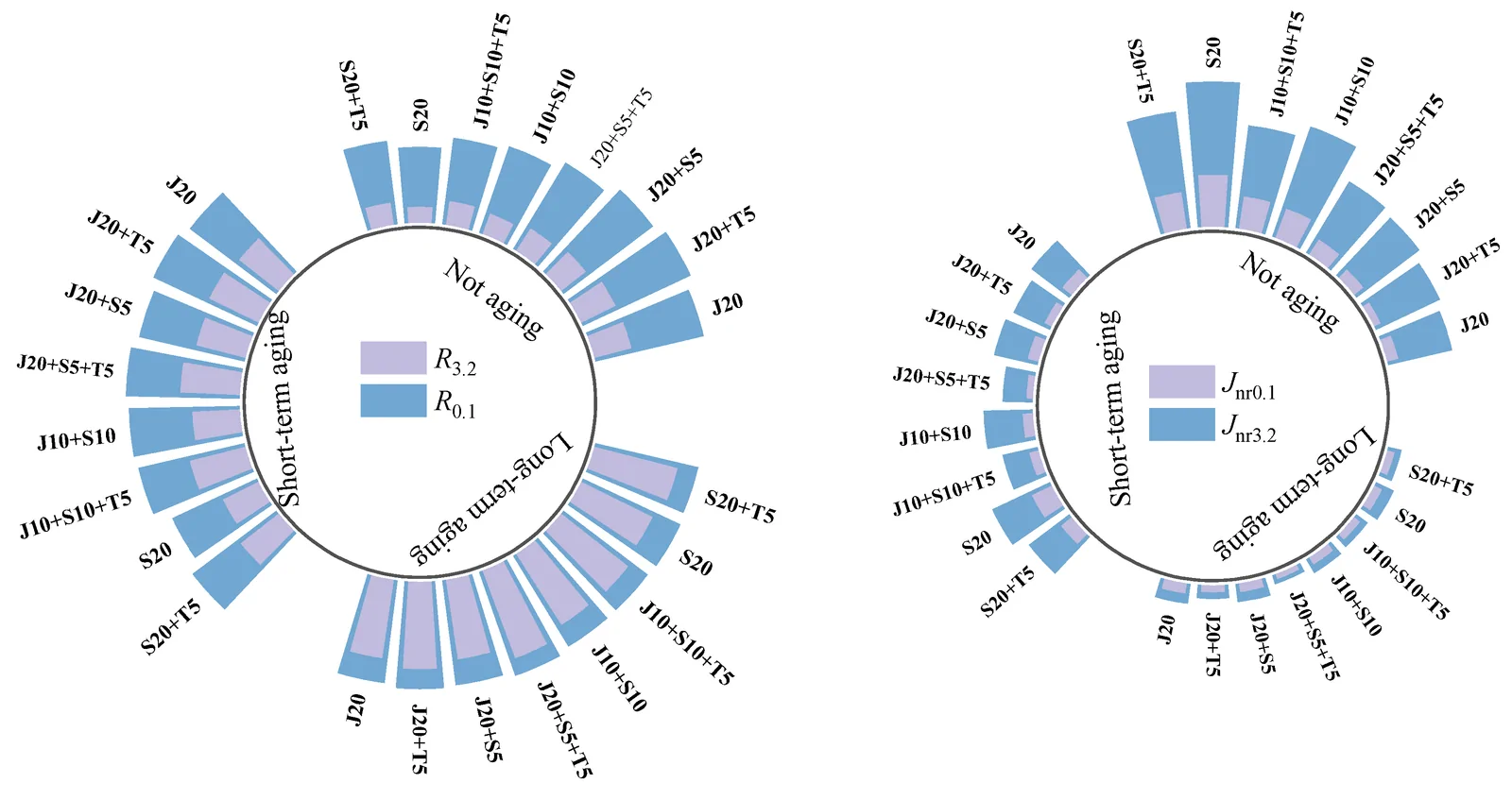
Creep recovery rate (*R*) and non-recoverable creep compliance (*J*_nr_) under different aging conditions.

To quantify the effect of the aging degree on the deformation resistance of the modified asphalt, the creep recovery rate *R* and non-recoverable creep compliance *J*_nr_ were introduced, and the relevant results are shown in Figure 7.

The effect of different crumb rubber types, TOR dosage, and aging degrees on the creep recovery rate (R) and non-recoverable creep compliance (J_nr_) of the modified asphalt was systematically analyzed. R decreased significantly with the increase in the stress level, whereas Jnr increased with the stress increase, and both were more sensitive to the material composition and aging degree under high stress levels. Under unaged conditions, the crumb rubber-modified asphalt (J20) showed the best elastic recovery capacity and the lowest non-recoverable deformation, whereas the R_3.2_ of the plasticized crumb rubber (S20) decreased significantly to 11.9% and J_nr3.2_ increased to 0.9032 kPa^−1^, indicating that the plasticization treatment improved compatibility but deteriorated the permanent deformation resistance. TOR can further optimize the J20 deformation resistance under unaged conditions, but the improvement effect on S20 is limited. With the increase in aging, the R_3.2_ of all specimens increased significantly, and J_nr3.2_ decreased remarkably. The R_3.2_ of S20 increased from 11.9% to 59.6%, and J_nr3.2_ decreased from 0.9032 kPa^−1^ to 0.1211 kPa^−1^. After long-term aging, the R_3.2_ of the mixed specimen (J10 + S10) reached 63.2%, and J_nr3.2_ was 0.0969 kPa^−1^, indicating that its high-temperature permanent deformation resistance (MSCR) was superior to that of the crumb rubber-modified asphalt. TOR enhanced the deformation resistance of all specimens after aging. After long-term aging, the R_3.2_ of J20 increased from 28.6% to 59.57%, and J_nr3.2_ decreased by 34.1%.

To further clarify how TOR improves the rheological properties of the aged composite binder, the recovery rates at 0.1 and 3.2 kPa were compared, and the stress sensitivity index ΔR = R_0.1_ − R_3.2_ was calculated. Under the unaged condition, the effect of TOR was not consistent; for example, ΔR increased from 55.0% for J20 to 61.26% for J20 + T5, while it slightly increased from 45.56% for S20 to 46.84% for S20 + T5. After short-term aging, TOR generally reduced ΔR for most systems, such as from 43.46% for J20 + S5 to 40.82% for J20 + S5 + T5 and from 47.76% for J10 + S10 to 39.03% for J10 + S10 + T5. After long-term aging, the beneficial effect of TOR became more evident. For J20, the addition of TOR increased R3.2 from 59.57% to 65.81% and decreased ΔR from 18.46% to 15.36%. For J20 + S5, R3.2 increased from 60.44% to 70.55%, and ΔR decreased from 19.31% to 12.54%. For S20, R3.2 increased from 59.59% to 64.33%, and ΔR decreased from 21.44% to 15.22%. These results indicate that TOR mainly improves the aged rheological performance by enhancing elastic recovery under high stress and reducing stress sensitivity, rather than by simply increasing the initial modulus. This can be attributed to the role of TOR in stabilizing the rubber–asphalt elastic network during aging.

In addition to R, the stress sensitivity of Jnr was also evaluated. To quantify this, the index J = (J_nr3.2_ − J_nr0.1_)/J_nr0.1_ was introduced. For J20, J decreased from 5.01 in the unaged state to 3.21 after TFOT and further to 1.03 after PAV. For S20, J changed from 1.81 to 1.96 and then to 1.38. After long-term aging, all binders had J values close to or below 1.4, indicating a much weaker stress dependence. TOR further reduced the stress sensitivity in most aged systems. For example, after PAV aging, J20 + S5 + T5 showed the lowest J, and J20 + T5 also had a low J of 0.98. These results indicate that aging hardens the binder and stabilizes the elastic network, thereby reducing the stress sensitivity of J_nr._ The addition of TOR helps maintain this stable elastic network after long-term aging, leading to lower non-recoverable deformation under high stress.

In general, the crumb rubber-modified asphalt exhibited excellent elastic recovery and permanent deformation resistance under both unaged and aged conditions. The plasticized crumb rubber showed significantly improved performance after aging, although its stress sensitivity was higher than that of J20 before aging. The composite blending of the two types of crumb rubber with TOR further optimized the rheological properties after aging, mainly by increasing R_3.2_ and reducing ΔR and J. This indicates that TOR helps stabilize the rubber–asphalt elastic network under high stress after long-term aging, providing a theoretical basis for the composition design of crumb rubber-modified asphalt in different service stages.

## 4. Conclusions

In this study, 40-mesh ordinary crumb rubber, plasticized crumb rubber, and TOR were added to SK90# base asphalt to prepare composite modified asphalt. The rheological behavior of the crumb rubber/plasticized crumb rubber composite modified asphalt under different aging degrees was studied through physical and rheological property tests. The specific conclusions are as follows:

(1) The incorporation of crumb rubber significantly enhanced the macroscopic hardness and high-temperature deformation resistance of asphalt. Plasticization improved the compatibility between crumb rubber and asphalt but reduced the viscosity and high-temperature modulus. TOR enhanced the resistance of the modified asphalt to external loading.
(2) G* and G*/sinδ decreased with increasing temperature and increased with prolonged aging time. Under the aging conditions used in this study, the plasticized crumb rubber-modified asphalt exhibited greater aging sensitivity in terms of high-temperature rheological properties, especially after long-term aging. This phenomenon may be related to changes in the crumb rubber structure during plasticization and an increase in oxidation sensitivity; however, the underlying molecular mechanism still requires further verification. The composite blending of the two types of crumb rubber balanced, to a certain extent, the high-temperature performance and aging sensitivity. TOR had a limited effect on reducing the aging sensitivity of plasticized crumb rubber asphalt.
(3) R decreased significantly with increasing stress level, while Jnr increased with increasing stress; both were more sensitive to material composition and aging degree under high stress. Under unaged conditions, J20 exhibited the best elastic recovery and the lowest unrecoverable deformation. For S20, R3.2 was only 11.9%, and Jnr3.2 was 0.9032 kPa^−1^, indicating that the plasticized system had relatively low resistance to permanent deformation before aging. After long-term aging, the J values of all asphalts were close to or below 1.4, indicating that the stress dependence was significantly weakened. TOR increased R3.2 in most systems and further reduced ΔR and J. These results indicate that TOR improved the elastic recovery under high stress after long-term aging and reduced the stress sensitivity.

This study is mainly based on laboratory accelerated aging tests, such as TFOT and PAV, which cannot fully reproduce the long-term aging behavior under the coupled effects of ultraviolet radiation, moisture, load, and other factors during actual pavement service. Future studies should focus on the performance evolution of crumb rubber-modified asphalt under coupled outdoor conditions, and combine gel permeation chromatography (GPC), atomic force microscopy (AFM), and other complementary techniques to verify and elucidate the evolution of molecular structure and micro-morphology during TOR cross-linking and aging. Such studies are necessary to confirm the proposed mechanisms and establish more reliable structure–property relationships.

## Figures and Tables

**Table 1:** Technical indicators of the materials used.

Material	Technical Indicators	Measured Value	Specification Requirement
SK90# asphalt	Viscosity at 135 °C	594 mPa·S	-
	Penetration (25 °C, 5 s, 100 g)/0.1 mm	89.8	80–90
	Penetration at 25 °C after TFOT/0.1 mm	48.5	-
	Penetration ratio of TFOT residue/%	54.01%	≥50
	Softening point/°C	44.6	≥42
	Softening point after TFOT/°C	50.1	
	Softening-point increment of TFOT residue/°C	5.5	
	Ductility at 15 °C, 5 cm/min/cm	>100	≥100
	Mass change after TFOT/%	0.4	≤±0.8
Crumb Rubber	Heating loss/%	0.8	≤1
	Ash content/%	6	≤10
	Iron content/%	0.01	≤0.05
	Fiber content/%	0	<1

Note: TFOT, thin film oven test.

**Table 2:** Basic properties of Vestenamer^®^ 8012.

Technical Indicators	Tested Results
Form	granular
Glass transition temperature/°C	–65
Melting point/°C	54
Density/(g/cm^3^)	0.91

**Table 3:** Sulfoxide index.

Sample	A_S=O_	AR	SI
J20	102.517	1350.304	0.075921
S20	94.569	1191.28	0.079384
T(J20)	101.582	1260.334	0.080599
T(S20)	112	1327.356	0.084378
P(J20)	95.81	684.582	0.139954
P(S20)	135.231	950.274	0.142307

## Data Availability

The original contributions presented in this study are included in the article. Further inquiries can be directed to the corresponding author.
